# Occurrence and Characterization of Graft-Transmissible Pathogens of Citrus in Orchards and Urban Citrus in Chile with a Focus on the *Apscaviroid nanocitri*

**DOI:** 10.3390/plants15172697

**Published:** 2026-09-02

**Authors:** Natalia Riquelme, Nicolás Quiroga, Claudio Henríquez, Pía Varas, Georgios Vidalakis, Camila Gamboa, Catalina Ferreira, Alan Zamorano, Nicola Fiore, Ximena Besoain

**Affiliations:** 1 Escuela de Agronomía, Pontificia Universidad Católica de Valparaíso, Casilla 4D, Quillota 2260000, Chile; natalia.riquelme@pucv.cl (N.R.); claudio.henriquez@pucv.cl (C.H.); pia.varas@pucv.cl (P.V.); catalina.ferreira.fs@gmail.com (C.F.); 2 Millennium Nucleus Bioproducts, Genomic and Environmental Microbiology (BioGEM), Avenida España 1680, Valparaíso 2390123, Chile; 3 Departamento de Sanidad Vegetal, Facultad de Ciencias Agronómicas, University of Chile, Santiago 8820808, Chile; nicolas.quiroga@uoh.cl (N.Q.); camila.gamboa@uchile.cl (C.G.); agezac@uchile.cl (A.Z.); 4 Department of Microbiology and Plant Pathology, University of California, Riverside, CA 92521, USA; georgios.vidalakis@ucr.edu

**Keywords:** *Apscaviroid nanocitri*, *Hostuviroid impedihumuli*, *Pospiviroid exocortiscitri*, biological indexing, mixed infections, urban citrus, epidemiology, citrus orchards

## Abstract

*Apscaviroid nanocitri* (citrus dwarfing viroid, CDVd) is a graft-transmissible pathogen that frequently coexists with other citrus viroids and affects tree vigor. This study evaluated the occurrence of citrus viroids in small growers and commercial orchards in Chile and characterized CDVd strains obtained from urban *Citrus* spp. trees, with unknown propagation history following the discontinuation of the national citrus certification program. A total of 301 samples from five citrus-producing regions were analyzed, revealing that CDVd is widely distributed and commonly associated with *Hostuviroid impedihumuli* (hop stunt viroid, HSVd). Single infections were the most frequent (41%), followed by double (7.3%) and triple infections (3.0%), indicating that mixed infections are common. CDVd strains from urban trees were characterized using biological indexing on ‘Etrog’ citron Arizona 861-S-1 indicator plants combined with RT-PCR detection and phylogenetic analysis. Symptom development and pathogen detection were evaluated at 6 and 18 months post-inoculation. All graft-inoculated plants developed symptoms consistent with viroid infection, whereas control plants remained asymptomatic. CDVd was consistently detected at 18 months but not at earlier stages, suggesting delayed systemic accumulation. Although *Closterovirus tristezae* (citrus tristeza virus, CTV) was present in the source plants, it was not detected in indicator plants at either time point, possibly associated with the higher temperatures required for viroid symptom development during bioindexing. The frequent occurrence of mixed infections highlights the epidemiological relevance of viroid interactions and supports the need for reinstatement and strengthening of the national citrus certification program.

## 1. Introduction

Grafting is fundamental to modern citrus production because the interaction between scion and rootstock determines key agronomic traits such as vigor, canopy architecture, yield, fruit quality, and management efficiency [1,2,3,4]. The extensive use of vegetative propagation and budwood exchange in citrus cultivation has historically facilitated the dissemination of graft-transmissible pathogens, including viruses and viroids [5,6,7,8]. In addition to graft transmission, citrus viroids can also be mechanically transmitted through contaminated pruning or cutting tools during routine horticultural practices [7,9].

Citrus certification programs have been implemented worldwide to prevent the dissemination of graft-transmissible pathogens using pathogen-free propagation material [10]. These programs are based on the sanitary selection, indexing, and maintenance of clean mother plants, and have proven effective in reducing the incidence of graft-transmissible diseases and improving productivity [11]. In Chile, a national citrus certification program was established and remained active for over a decade, contributing to the production and distribution of sanitized plant material; however, its discontinuation in 2010 [12] may have facilitated the re-emergence and spread of viroids under current production systems. While participation in the previous Chilean program was voluntary, the literature emphasizes the necessity of mandatory certification to fully safeguard the industry, especially because voluntary programs are particularly inadequate against insect-vectored pathogens [8,10,13].

In the absence of citrus certification programs, citrus trees frequently harbor multiple graft-pathogens simultaneously, resulting in complex infection profiles within individual hosts [8]. Recent epidemiological surveys of citrus orchards have confirmed that several viroid species are commonly detected in mixed infections within the same plant [8]. This infectious complexity can affect pathogen accumulation, symptom expression, and overall plant performance [14,15,16,17,18,19]. Citrus hosts several viroid species that can have significant biological and agronomic impacts, including *Pospiviroid exocortiscitri* (citrus exocortis viroid, CEVd), *Hostuviroid impedihumuli* (hop stunt viroid, HSVd), *Apscaviroid curvifoliumcitri* (citrus bent leaf viroid, CBLVd), *Apscaviroid nanocitri* (citrus dwarfing viroid, CDVd), *Cocadviroid rimocitri* (citrus bark cracking viroid, CBCVd), *Apscaviroid epsiloncitri* (citrus viroid V, CVd-V), *Apscaviroid zetacitri* (citrus viroid VI, CVd-VI), and *Apscaviroid etacitri* (citrus viroid VII, CVd-VII) [8]. Among these pathogens, CDVd is of particular interest due to its effects on vegetative vigor and tree architecture. CDVd variants can cause different symptom severities in indicator plants. These range from mild to severe phenotypes, with a clear association between leaf symptoms and reduced plant growth [20,21]. Several CDVd sequence variants have been described and grouped into distinct phylogenetic lineages: IIIa, IIIb, and IIIc. These differ in sequence composition and biological characteristics [20,22]. The impact of CDVd varies on the scion–rootstock combination [8,23]. In some systems, CDVd reduces growth and canopy size. For this reason, it has been evaluated experimentally as a biological dwarfing agent for high-density citrus orchards, especially with trifoliate orange [*Poncirus trifoliata* (L.) Raf.] and related hybrid rootstocks [5,8,19,20,21,24,25].

In Chile, graft-transmissible citrus pathogens have been historically documented through biological indexing across various producing regions [26,27]. Subsequent studies combining biological indexing with sequential polyacrylamide gel electrophoresis (sPAGE) confirmed the presence of citrus viroids associated with cachexia disease affecting mandarin (*Citrus reticulata* Blanco) and lemon [*C. × limon* (L.) Osbeck] trees [28]. Symptoms consistent with this disease, including wood pitting, gum impregnation, and severe chlorosis, were observed in infected plants. Based on its electrophoretic mobility and biological behavior, the causal agent was identified as the cachexia-inducing variant (CVd-IIb) of the species HSVd [28]. Furthermore, biological indexing identified exocortis symptoms in lemon trees grafted onto *P. trifoliata* rootstocks, providing early evidence of the occurrence of CEVd in the country [26]. These findings demonstrated that viroid-associated diseases have been present in Chilean citrus systems for decades, affecting multiple host species and highlighting the early establishment of graft-transmissible pathogens under local conditions.

Subsequent studies in Chile have explored diagnostic approaches integrating biological indexing with molecular detection, enabling the identification of citrus viruses and viroids in indicator plants [29]. In this context, the development of reverse transcription polymerase chain reaction (RT-PCR) methodologies has significantly improved the sensitivity and reliability of viroid detection, allowing the amplification and characterization of multiple viroid species from infected plant tissues [30]. Surveys in other citrus-producing countries have confirmed that CDVd is frequently found in commercial citrus orchards and is commonly associated with HSVd, indicating that mixed infections are prevalent under local conditions [31,32,33,34].

In addition to viroids, *Closterovirus tristezae* (CTV), one of the most economically important graft-transmissible pathogens affecting citrus worldwide, is also present in Chile [26,29,35,36]. Virus–viroid interactions may influence pathogen accumulation, and under certain host conditions, CTV has been shown to enhance the accumulation of CDVd through virus-encoded RNA silencing suppressors [37].

Based on this background, this study conducted a survey to evaluate the current occurrence and detection frequencies of citrus viroids and CTV in commercial citrus orchards in Chile, eight years after the discontinuation of the national certification program. We additionally chose as a case study to characterize Chilean CDVd strains obtained from urban *Citrus* spp. trees, including sweet orange (*Citrus* × aurantium var. *sinensis* L.) and lemon, using biological indexing in ‘Etrog’ citron (*Citrus medica* L.) Arizona 861-S-1 indicator plant, combined with RT-PCR detection and molecular characterization through phylogenetic analysis. To further assess the behavior of the CDVd strains under controlled conditions, molecular detection was performed at two points to evaluate the temporal dynamics of viroid accumulation following graft inoculation. Our findings are discussed in relation to pathogen detection assays, viroid biology, symptom expression and epidemiology, and the critical importance of reinstating citrus certification strategies to ensure the sanitary quality of propagation material and to mitigate the impact of mixed infections in orchards and urban ornamental citrus plantings in Chile.

## 2. Results

### 2.1. Occurrence of Citrus Viroids and CTV in Chilean Citrus Orchards

A survey of citrus orchards in Chile, based on the analysis of 301 samples collected across five citrus-producing regions, revealed that the analyzed graft-transmissible pathogens are widely distributed in commercial orchards. Among citrus viroids, HSVd and CDVd showed the highest number of positive samples, 29.6% and 23.9%, respectively. CEVd was detected at a low frequency (2%) in the surveyed trees, whereas CTV was detected in 17.9% of samples (Table 1).

These pathogens were detected across multiple citrus species, including mandarins, sweet oranges, lemons, clementines (*Citrus* × *aurantium* L.), and grapefruit (*Citrus* × *aurantium* L. var. *racemosa* (Risso) ined.), with variable percentages of positive samples among hosts. HSVd showed the highest number of infected samples in mandarins (44.3%) and sweet oranges (40.8%). CDVd was also frequently detected in mandarins (29.6%) and sweet oranges (33.8%). In contrast, CEVd was detected at a low frequency across species, while CTV showed a moderate percentage of positive samples, particularly in lemons (22.1%).

Mixed infections involving the analyzed pathogens were identified in a subset of the surveyed trees (Table 2). Among the analyzed samples, single infections were the most frequent (125/301), followed by double (22/301) and triple (9/301), as shown in Table 3. Among mixed infections, the CDVd + HSVd combination was the most frequently detected, occurring in 35 samples (11.6%). This combination was observed across multiple citrus species, predominantly in mandarins (78.3%), followed by clementines (60.0%), lemons (12.5%), and grapefruits (100%). Triple infections were less frequent and included combinations such as CTV + HSVd + CDVd (8 samples; 2.7%) and CTV + HSVd + CEVd (1 sample; 0.3%). These were detected mainly in sweet orange and lemon, with only occasional cases observed in clementine and mandarin.

### 2.2. Symptom Development in ‘Etrog’ Citron Following Graft Inoculation

Successful graft unions were established in all indicator plants, ensuring vascular connectivity between donor and recipient tissues.

**Figure 1:**
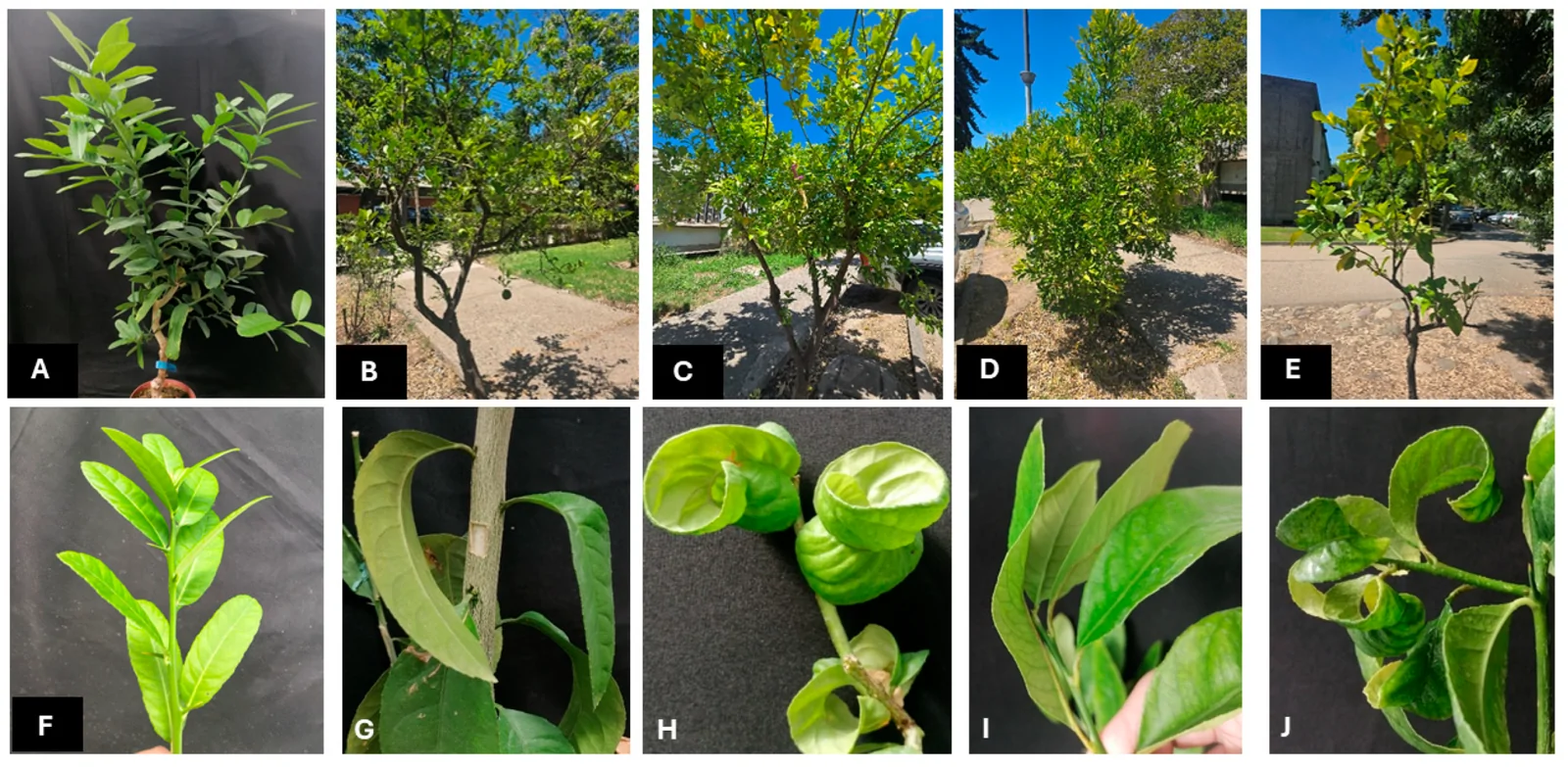
Symptomatology observed in ‘Etrog’ citron Arizona 861-S-1 plants following patch graft inoculation with samples from urban *Citrus* spp. plants. (**A**): Healthy ‘Etrog’ citron Arizona 861-S-1 indicator plant (negative control). (**B**–**E**): Urban citrus donor plants of unknown cultivar and propagation history sampled in this study. (**B**): P1 (sweet orange); (**C**): P21 (lemon); (**D**): P28 (sweet orange); (**E**): P37 (lemon). (**F**): Leaves of the healthy ‘Etrog’ citron Arizona 861-S-1 indicator plant. (**G**–**J**): ‘Etrog’ citron Arizona 861-S-1 indicator plants, photographed 18 months after inoculation, showing symptoms following inoculation with different citrus strains. (**G**): Plant inoculated with sample P1, showing leaf bending due to petiole and midrib necrosis consistent with CDVd infection. (**H**): Plant inoculated with sample P21, exhibiting leaf curling symptoms consistent with mixed infection by CDVd, HSVd, and CEVd. (**I**): Plant inoculated with sample P28, displaying leaf bending consistent with mixed infection by CDVd and HSVd. (**J**): Plant inoculated with sample P37, exhibiting leaf-curling symptoms consistent with mixed infection by CDVd, HSVd, and CEVd.

From the initial set of 13 graft-inoculated indicator plant groups, four strains (P1, P21, P28, and P37; see Figure 1) were selected for further analysis following symptom development and subsequent molecular confirmation of viroid infection.

Eighteen months post-inoculation, only the indicator plants grafted with viroid-infected scions developed symptoms (Figure 1); healthy controls remained asymptomatic (Figure 1A,F). The ornamental citrus donor plants used for graft inoculation are shown in Figure 1B–E, and their viroid and virus status is summarized in Table 4. Plants inoculated with sample P1 showed mild to moderate petiole and midrib necrosis (Figure 1G). Sample P21 induced severe leaf curling (Figure 1H). P28 caused leaf bending and moderate distortion of the leaf blade (Figure 1I). Plants inoculated with P37 exhibited pronounced leaf curling, similar in severity to P21 (Figure 1J).

These symptom patterns were associated with the viroid composition detected in each sample, with more severe leaf curling observed in samples presenting mixed infections (see Section 2.4).

### 2.3. Temporal Dynamics of Viroid Accumulation and Detection by RT-PCR in Indicator Plants

Molecular detection by RT-PCR revealed temporal variation in the presence of infectious agents in indicator plants (Table 4). At 6 months post-inoculation, CDVd was detected only in plants inoculated with strain P28. No CDVd was detected in P1, P21, or P37, and none of the samples tested positive for other viroids or CTV at this time point.

At 18 months post-inoculation, CDVd was detected in all inoculated plants (P1, P21, P28, and P37), indicating progressive systemic accumulation. In contrast, CTV was not detected in any indicator plant at either time point.

### 2.4. Detection of Mixed Viroid Infections in Indicator Plants

Analysis of viroid composition at 18 months post-inoculation revealed mixed infections in several indicator plants (Table 4). Sample P1 showed exclusive detection of CDVd, whereas P28 exhibited a mixed infection of CDVd and HSVd. In contrast, samples P21 and P37 showed more complex infection profiles, with the simultaneous detection of CDVd, HSVd, and CEVd.

Comparison with the RT-PCR results obtained from source plants indicated that, after biological index analysis, three viroid species were detected after 18 months in samples P21 and P37. However, only CDVd was consistently detected in all samples. These findings confirm that mixed viroid infections were successfully transmitted and maintained under biological indexing conditions.

### 2.5. Phylogenetic Relationships of Chilean CDVd Strains

**Figure 2:**
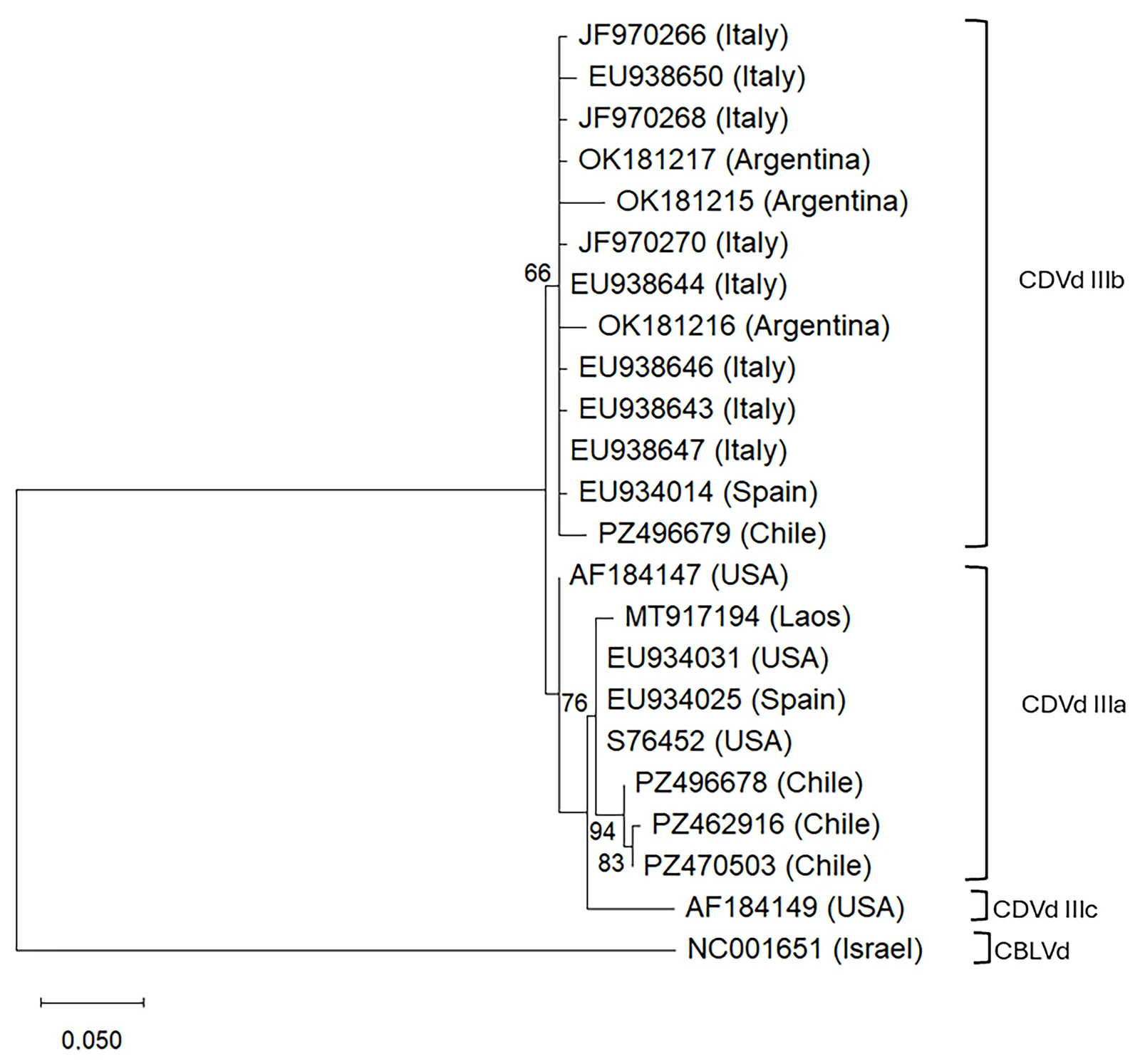
Maximum likelihood phylogenetic tree inferred from complete CDVd genome sequences obtained in this study together with representative reference sequences retrieved from GenBank (Supplementary Table S1). Bootstrap support values (>50%) based on 1000 replicates are shown at the nodes. CDVd phylogenetic groups (IIIa–IIIc) are indicated on the right. The tree was rooted using CBLVd (Acc. Number: NC_001651) as the outgroup.

Phylogenetic analysis was performed to determine the relationship between the CDVd strains obtained in this study and representative reference sequences retrieved from GenBank (Figure 2). The Maximum Likelihood tree resolved the sequences into three major CDVd variant groups (IIIa, IIIb, and IIIc), as previously described [38,39].

Three Chilean strains (P1, P21, and P28) clustered within the CDVd IIIa group, along with representative reference sequences such as EU934031, EU934025, MT917194, and S76452. In contrast, strain P37 grouped within the CDVd IIIb clade, along with representative reference sequences such as EU938650, JF970270, and AF184147 (Supplementary Table S1). These results indicate the presence of at least two CDVd variant groups among the Chilean strains analyzed in this study. The inclusion of CBLVd as an outgroup clearly separated the CDVd sequences from the ingroup, confirming the phylogenetic placement of the analyzed strains.

## 3. Discussion

The present study integrates field survey data from commercial citrus orchards in Chile with the biological and molecular characterization of CDVd strains obtained from urban citrus plants. The survey results demonstrate that CDVd is widely distributed in Chilean citrus orchards and frequently occurs in mixed infections, particularly in association with HSVd. The biological characterization further confirms that CDVd is readily transmitted by graft inoculation and consistently detected under biological indexing conditions following extended incubation periods, as previously reported [7,9,18,40,41]. In addition, the detection of CDVd in association with other viroids, including HSVd and CEVd, indicates that mixed infections are a defining feature of these strains, as reported from other citrus-producing countries that lack mandatory national citrus certification programs, such as Greece, Palestine, Laos, Japan, Iran, and Pakistan [6,10,31,33,42,43,44]. Notably, the concordance between the mixed infection profiles observed under controlled indexing conditions and those detected in commercial orchards suggests that these viroid associations are consistently detected across both experimental and field conditions.

The survey was designed as a targeted phytosanitary assessment rather than a random epidemiological survey. Consequently, sampling was preferentially directed toward trees showing symptoms potentially associated with graft-transmissible pathogens, whereas asymptomatic trees were included only when no symptomatic plants were present in an orchard. Therefore, the percentages of positive samples reported in this study should not be interpreted as estimates of pathogen prevalence in the Chilean citrus industry, but rather as an indication of the relative frequency with which these pathogens were detected among the surveyed trees. Nevertheless, together, these findings support the view that urban citrus plantings, particularly those with unknown propagation history, may represent epidemiologically relevant reservoirs contributing to the maintenance and dissemination of diverse viroid populations in Chilean citrus production systems.

Variability in symptom severity among strains indicates that biological responses are not solely determined by the presence of individual viroids but rather reflect the composition and interaction of viroid populations within each strain. This interpretation is consistent with previous studies showing that citrus samples frequently contain multiple viroids and that symptom expression in ‘Etrog’ citron depends on both viroid identity and interactions among co-infecting agents [15,16,17,45]. In particular, mixed infections have been shown to result in synergistic effects, leading to increased symptom severity or the expression of symptoms associated with more virulent viroids, especially under biological indexing conditions [17]. Notably, at least one sample contained only CDVd, and the symptoms observed in the corresponding indicator plants were consistent with those previously described for this viroid, supporting its direct contribution to the observed phenotype [16]. These findings reinforce the value of biological indexing as a functional approach to assessing the composite biological effects of complex viroid populations rather than individual pathogens [3].

Molecular analyses revealed a temporal pattern of pathogen detection, with CDVd detected only in a subset of plants at six months post-inoculation but consistently detected in all indicator plants at eighteen months. This delayed detection may reflect differences in replication dynamics and systemic movement following grafting, as previously reported for citrus viroids [7,9,42]. These results highlight that viroid accumulation to detectable levels may require extended incubation periods under biological indexing conditions, likely reflecting progressive systemic colonization of host tissues. This temporal dynamic has important implications for diagnostic sensitivity and supports the continued use of prolonged indexing periods in citrus certification programs.

Mixed infections involving CDVd, HSVd, and CEVd were identified in several graft-inoculated indicator plants, consistent with the presence of complex viroid populations in the donor plants. A limitation of this study is that HSVd and CEVd were not included in the original molecular testing of the donor plants; therefore, their presence at the time of graft collection could not be confirmed. Retrospective testing was not considered informative because the donor trees remained under urban conditions after sampling and were continuously exposed to natural sources of viroid infection. Nevertheless, all indicator plants originated from the same ‘Etrog’ citron Arizona 861-S1 source, and the absence of symptoms and negative molecular results in the non-inoculated controls suggests that latent infections in the indicator plants or cross-contamination are unlikely.

Such mixed infections in citrus have been extensively documented [3,15,16], and recent studies confirm the high complexity of viroid communities in citrus hosts. For example, simultaneous infections involving multiple viroids, including HSVd, CDVd, CBLVd, CEVd, and CBCVd, have been reported in kumquat (*Fortunella* spp.) [44], while additional viroid species such as CVd-VII continue to expand the known diversity of citrus viroids [46,47]. These findings support the view that citrus trees commonly harbor complex viroid populations rather than single infections, especially in the absence of a mandatory citrus certification program.

Phylogenetic analysis placed the four Chilean CDVd strains within two previously described CDVd variant groups (IIIa and IIIb), supporting the occurrence of at least two distinct CDVd lineages among the strains analyzed in this study. To provide a broader regional context, representative reference sequences from neighboring Argentina, together with representative strains from other geographic origins, were included in the phylogenetic reconstruction. The resulting topology was consistent with the previously described CDVd phylogenetic groups [20,22]. The assignment of the Chilean strains to groups IIIa and IIIb was supported by moderate to high bootstrap values, providing confidence in their phylogenetic placement. However, deeper relationships among the major CDVd groups remained less well resolved. Because only four complete Chilean CDVd genome sequences were available for phylogenetic analysis, these findings should be interpreted as evidence that the Chilean strains belong to two CDVd phylogenetic groups (IIIa and IIIb) previously described for this viroid, rather than as a comprehensive assessment of the genetic diversity of Chilean CDVd populations. Nevertheless, the occurrence of strains belonging to different phylogenetic groups suggests that more than one CDVd lineage is present in Chile. Additional complete genome sequences from different production areas will be necessary to better understand the origin and evolutionary relationships of these lineages.

Mixed viroid infections are a common feature of citrus pathosystems and can influence pathogen accumulation and symptom expression through interactions involving RNA–RNA dynamics and host regulatory pathways [14,16,25,43,48,49,50]. In addition, interactions between viruses and viroids may further modulate pathogen dynamics, as CTV has been shown to enhance the accumulation of CDVd through virus-encoded RNA silencing suppressors under specific hot conditions [37]. Experimental evidence also suggests that co-infection by CEVd and HSVd may result in coordinated accumulation patterns and similar tissue localization, indicative of non-competitive or potentially cooperative interactions during systemic infection [51]. Consistent with previous findings, the survey conducted in this study indicates that mixed infections involving CDVd and HSVd are the most frequent in Chilean citrus orchards. More complex infection profiles, including triple infections with CTV, appear less common. The predominance of CDVd + HSVd across different citrus hosts suggests that this combination represents a stable infection profile under field conditions [5,15,16,31].

A notable finding was the absence of CTV detection in indicator plants, despite its presence in some donor plants. CTV is a phloem-restricted virus with systemic distribution, although viral titers may vary depending on tissue type and environmental conditions [52,53,54]. The lack of detection in indexed plants likely reflects experimental conditions rather than true absence. Specifically, the elevated temperatures (28–32 °C) continuously maintained in the greenhouse to optimize viroid replication and symptom expression in citron are known to significantly restrict CTV replication and systemic translocation, leading to a marked depletion of in vivo viral titers as previously documented [52]. Indeed, this marked in vivo thermosensitivity of CTV at warm temperatures is the biological basis of heat-therapy sanitization protocols used globally to eliminate closteroviruses from budwood [11,40], whereas citrus viroids remain highly stable and replicate actively under these thermal conditions [15]. Furthermore, the use of ‘Etrog’ citron Arizona 861-S-1 as an indicator host likely also contributed to these observations. This genotype is highly sensitive to citrus viroids and widely used for their biological amplification and indexing [7,9], but is not the optimum or preferred bioindicator for the detection of CTV [7,18].

The detection of CDVd in urban citrus highlights the epidemiological significance of non-commercial hosts. In this study, the source plants corresponded to urban citrus trees of unknown cultivar, rootstock, and propagation history, reflecting the limited traceability typical of such plantings. These trees, frequently propagated vegetatively and when maintained outside certification systems, may act as reservoirs of graft-transmissible pathogens and contribute to their dissemination into commercial orchards. High-throughput sequencing studies have revealed complex virome compositions in citrus and underscore the role of propagation material in pathogen spread [55]. Previous work has similarly emphasized the importance of plant material exchange in the global dissemination of citrus viroids [7,9,46].

These findings expand the current understanding of CDVd in Chile. Although citrus viroids have been previously reported in the country [27], CDVd has not yet been biologically characterized under local conditions. The integration of biological indexing and field survey data supports the widespread occurrence of CDVd and its frequent association with HSVd in the field. Future studies incorporating comparative genomics and expanded phylogenetic analyses will be necessary to determine whether these strains represent novel variants or belong to established global lineages.

In the global hierarchy of citrus pathogens, CTV is recognized as the most devastating, responsible for the loss of over 50 million trees [8,10,52]. While CTV and the disease known as “huanglongbing” represent primary threats to tree survival, citrus viroids represent an equally significant, though more insidious, threat through chronic decline and sustained productivity losses [10,44,56]. The economic repercussions are profound; for instance, a study in Belize documented that viroid-infected trees yielded only 10.2 kg of fruit compared to the 40.8 kg produced by healthy trees, with losses estimated at $8685 USD per hectare when adjusted for inflation [10,56]. Furthermore, viroid-induced bark cracking predisposes trees to lethal *Phytophthora* spp. infections [21,56]. The identification of CDVd in Chilean urban citrus is of critical concern, as similar ornamental reservoirs in Europe have resulted in eradication costs exceeding €35 million [57]. In standard-density plantings, uncontrolled CDVd infection can reduce yields by 32.7% and canopy volume by approximately 50%, a risk that is particularly acute for the Chilean industry given its reliance on trifoliate and hybrid rootstocks that can suffer yield losses of up to 69% [21,24,39]. This economic burden is further compounded by CTV, as interactions between these pathogens can lead to significantly higher viroid titers, further compromising the sanitary status and productivity of the trees [37].

In summary, this study provides evidence of the biological transmission and molecular detection of CDVd from urban citrus sources and highlights the relevance of mixed infections in citrus viroid pathosystems. The results obtained from commercial orchards demonstrate that CDVd is widely distributed in productive citrus systems in Chile and commonly occurs as part of mixed infection complexes, particularly in association with HSVd, which may influence disease expression and pathogen dynamics under field conditions. These findings underscore the urgent need to reinstate the use of certified, pathogen-free propagation material in Chilean citrus production systems. Although initial regulatory efforts have been implemented to promote the use of sanitized plant material in nurseries, more comprehensive certification programs are required to ensure that propagation material used in commercial orchards and urban settings is free of viruses and viroids. Recent detection of citrus viroids in asymptomatic commercial and germplasm material further highlights the limitations of symptom-based surveillance and supports the implementation of sensitive molecular screening of propagation material [58]. Furthermore, the long-term impact of these mixed infections should be investigated in greater detail, specifically evaluating their effects on the scion–rootstock combinations currently used in Chile.

## 4. Materials and Methods

### 4.1. Survey of Citrus Orchards in Chile

A survey was conducted between 2017 and 2021 as a complementary epidemiological component to contextualize the biological characterization of CDVd strains. A total of 301 samples were collected from 92 orchards located across five major citrus-producing regions in Chile: Tarapacá, Coquimbo, Valparaíso, Metropolitana, and O’Higgins. The surveyed area accounted for approximately 7% of the country’s total citrus-growing area and was designed to capture a representative distribution of citrus production. Sampling was performed over three periods according to regional production cycles. The number of samples collected per region was determined based on cultivated area, following a proportional sampling strategy of approximately one sample per 100 hectares [59]. Each sample corresponded to a single tree. Plant material was collected from multiple citrus species, including sweet orange, mandarin, lemon, clementine, and grapefruit.

Sampling was primarily directed toward trees exhibiting symptoms potentially associated with graft-transmissible pathogens, including chlorosis, leaf deformation, or general decline. In orchards where no symptoms were observed, asymptomatic trees were randomly selected. This sampling strategy may have biased the dataset toward symptomatic infections and should therefore be considered when interpreting the percentages of positive samples reported in this study. From each sampled tree, four shoots (~15 cm in length) were collected from different sectors of the canopy to ensure representative sampling. Samples were transported under refrigerated conditions and stored at 4 °C until nucleic acid extraction.

### 4.2. Source Plants and Graft Inoculation

Initially, a collection of 45 citrus trees established along an urban street at the Antumapu Campus, University of Chile, previously analyzed for the presence of viruses and viroids, was considered. From this population, 13 trees displaying symptoms putatively associated with graft-transmissible pathogens were selected for sampling. Scions (~15 cm in length) were collected from each selected tree using pruning tools disinfected with 1% sodium hypochlorite between cuts to prevent cross-contamination. Each sample was placed in an individually labeled plastic bag and transported on ice to the Plant Pathology Laboratory at the Escuela de Agronomía, Pontificia Universidad Católica de Valparaíso (PUCV).

Biological indexing was performed using ‘Etrog’ citron Arizona 861-S-1 grafted onto rough lemon (*Citrus* × *granulata* Raf.) rootstock as indicator plants, previously confirmed to be free of viruses and viroids. For each of the 13 source plants, three indicator plants were graft-inoculated using two patch buds per plant. Negative control plants were grafted using material obtained from a healthy citrus plant.

All plants were maintained in a temperature-controlled greenhouse at 28–32 °C for 18 months [7,60]. Indicator plants were periodically evaluated for symptom development, and samples were collected at predefined time points for subsequent molecular analyses.

### 4.3. Nucleic Acid Extraction

Total RNA was extracted from 100 mg of pooled midrib and green bark tissues using the RNeasy Plant Mini Kit (Qiagen, Germany), following the manufacturer’s protocol.

### 4.4. Molecular Detection of Citrus Viroids and CTV

#### 4.4.1. Detection of CDVd

Detection of CDVd was performed by simplex one-step RT-PCR using the primer pair described by Ito et al. [33] (Table 5). Each 20 μL RT-PCR reaction contained 10 μL GoTaq^®^ Green Master Mix (2×; Promega, Madison, WI, USA), 0.4 μL GoScript™ Reverse Transcriptase (Promega), 1.25 μM of each primer, and 0.5 μL total RNA. Thermocycling conditions were reverse transcription at 37 °C for 15 min; initial denaturation at 94 °C for 5 min; 35 cycles of 94 °C for 30 s, 60 °C for 30 s, and 72 °C for 30 s; final extension at 72 °C for 7 min.

#### 4.4.2. cDNA Synthesis

For cDNA synthesis, 2 μL of total RNA was combined with 1 μL random primers (Thermo Scientific (Waltham, MA, USA); 0.2 μg μL^−1^), 1 μL dNTPs (10 μM each), and 7 μL nuclease-free water (total 11 μL). The mixture was heated at 65 °C for 5 min, then chilled on ice for 5 min. Next, 8 μL RT master mix was added (2 μL M-MuLV Reaction Buffer, 1 μL M-MuLV Reverse Transcriptase, 0.2 μL RNase Inhibitor, 4.8 μL nuclease-free water; all from New England Biolabs (Ipswich, MA, USA)). Each 20 μL reaction was mixed and incubated at 42 °C for 60 min, then inactivated at 70 °C for 10 min. The resulting cDNA was stored at −20 °C until further analysis.

#### 4.4.3. Detection of CEVd, HSVd, and CTV

PCR amplification of CEVd and HSVd was performed using SapphireAmp^®^ Fast PCR Master Mix (Takara Bio (Kusatsu, Japan)). Each 20 μL reaction contained 5.5 μL Master Mix, 1 μL each primer (10 μM; see Table 5), and 13.5 μL nuclease-free water, with cDNA as the template. Thermocycling conditions were 94 °C for 3 min; 40 cycles of 94 °C for 1 min, 55 °C for 1 min, 72 °C for 1 min; final extension at 72 °C for 7 min.

PCR amplification of CTV was performed using SapphireAmp^®^ Fast PCR Master Mix (Takara Bio). Each 22 μL reaction included 12.5 μL Master Mix, 1 μL each of primer (10 μM) (Table 4), and 7.5 μL nuclease-free water, with cDNA as template. Thermocycling conditions were 94 °C for 2 min; 30 cycles of 94 °C for 30 s, 55 °C for 30 s, 72 °C for 45 s; final extension at 72 °C for 5 min.

PCR products were resolved by electrophoresis on a 1.5% (*w*/*v*) agarose gel stained with GelRed^®^, Biotum (Fremont, CA, USA) and visualized under UV light. Amplicons were purified and sequenced by Macrogen (Seoul, Republic of Korea). Sequence identities were verified using BLASTn against the NCBI nucleotide database.

### 4.5. Phylogenetic Analysis

To determine the phylogenetic placement of the CDVd strains obtained in this study, complete CDVd genome sequences were compared with representative reference sequences retrieved from the NCBI GenBank database. Representative strains of the previously described CDVd phylogenetic groups IIIa, IIIb, and IIIc were included, together with all complete CDVd genome sequences available from neighboring Argentina to provide a broader regional phylogenetic context. Citrus bent leaf viroid (CBLVd; NC_001651) was included as the outgroup because of its phylogenetic proximity within the genus Apscaviroid. Sequences were aligned using the MUSCLE algorithm implemented in MEGA version 12. The best-fitting nucleotide substitution model was selected using the Bayesian Information Criterion (BIC) implemented in MEGA12. Phylogenetic reconstruction was subsequently performed using the Maximum Likelihood method under the Kimura 2-parameter model with a proportion of invariant sites (K2 + I). Statistical support for the inferred topology was assessed using 1000 bootstrap replicates.

### 4.6. Detection Frequencies and Infection Profiles

Percentage of positive samples was calculated as the proportion of RT-PCR positive samples relative to the total number of samples analyzed (n = 301). For each pathogen and infection profile, the number of positive samples was divided by the total number of samples analyzed and multiplied by 100. Single and mixed infections (double and triple) were quantified using the same approach. Only samples testing positive by RT-PCR were considered for calculations of the percentage of positive samples. The percentage of positive samples and infection profiles (single and mixed) were expressed as absolute frequencies (number of positive and negative samples) and relative frequencies (percentages) to describe the baseline sanitary status of the orchards surveyed.

## 5. Conclusions

This study demonstrates that CDVd was frequently detected in the surveyed Chilean citrus production systems and commonly occurred in mixed infections, particularly in association with HSVd. The integration of field survey data with biological indexing and molecular detection revealed diverse infection profiles involving citrus viroids and CTV.

Biological indexing confirmed CDVd transmissibility and highlighted the importance of extended incubation periods for reliable detection, reflecting the temporal dynamics of viroid accumulation. The presence of mixed viroid populations was associated with variability in symptom expression, supporting the potential role of pathogen interactions in shaping disease phenotypes.

Furthermore, the detection of CDVd in urban citrus plants underscores the epidemiological relevance of non-commercial hosts as potential reservoirs of graft-transmissible pathogens. These findings emphasize the need to consider both commercial and non-commercial citrus populations in phytosanitary management strategies and certification programs.

Overall, this study provides new insights into the occurrence of graft-transmissible citrus pathogens and the biological behavior and epidemiological significance of CDVd in Chile, contributing to a better understanding of viroid pathosystems and emphasizing the importance of mandatory national citrus certification programs based on pathogen-tested propagation material.

## Figures and Tables

**Table 1:** Frequency of detection of viroids and CTV in Chilean citrus orchards.

Pathogen	Number of Positive Samples (n = 301)	Percentage of Positive Samples
CTV	54	17.9
HSVd	89	29.6
CEVd	6	2.0
CDVd	72	23.9

**Table 2:** Mixed infections involving CDVd, HSVd, CEVd, and CTV detected in Chilean citrus orchards.

Infection Profile	Number of Positive Samples/Total Samples Analyzed	Percentage of Positive Samples
CDVd + HSVd	35/301	11.6
CTV + HSVd + CDVd	8/301	2.7
CTV + HSVd + CEVd	1/301	0.3

**Table 3:** Distribution of infection types (single, double, and triple infections) among surveyed citrus trees from Chilean orchards.

Citrus Species	Collected Samples	Negative Samples	Type of Infection *		
			Simple	Double	Triple
Mandarins	88	11	51	18	0
Sweet Orange	71	20	26	0	6
Lemons	86	37	33	1	2
Clementines	46	27	10	3	1
Grapefruit	10	4	5	0	0
**Total**	**301**	**99**	**125**	**22**	**9**

* Positive samples were defined based on the detection of CTV, HSVd, CEVd, and CDVd. Negative samples are those that test negative for all analyzed pathogens.

**Table 4:** Reverse transcription polymerase chain reaction (RT-PCR) detection of viroids and CTV in source plants and ‘Etrog’ citron Arizona 861-S-1 indicator plants graft-inoculated with ornamental citrus strains.

Sample	Detection in Source Plants By RT-PCR Analysis				Detection at 6/18 Months Post-Inoculation **			
	CTV	HSVd	CEVd	CDVd	CTV	HSVd	CEVd	CDVd
P1	+	NT	NT *	−	−/−	−/−	−/−	−/+
P21	−	NT	NT	+	−/−	−/+	−/+	−/+
P28	−	NT	NT	+	−/−	−/+	−/−	+/+
P37	−	NT	NT	+	−/−	−/+	−/+	−/+

* NT: not tested. ** −/+, detection result at 6 and 18 months post-inoculation, respectively (−, not detected; +, detected).

**Table 5:** Primers used for viroids and CTV detection.

Marker	Sequence of Primers (5′→3′)	Reference
CDVd_F	CTCCGCTAGTCGGAAAGACTCCGC	[52]
CDVd_R	TCACCAACTTAGCTGCCTTCGTC	
CEVd_F	GGAAACCTGGAGGAAGTCGAG	[30]
CEVd_R	CCGGGGATCCCTGAAGGACTT	
HSVd_F	GGCAACTCTTCTCAGAATCCAGC	[61]
HSVd_R	CCGGGGCTCCTTTCTCAGGTAAGT	
CTV_F	ATGGACGACGAAACAAAGAAATTG	[62]
CTV_R	TCAACGTGTGTTGAATTTCCCA	

## Data Availability

The original contributions presented in this study are included in the article/Supplementary Materials. Further inquiries can be directed to the corresponding authors.

## Supplementary Materials

The following supporting information can be downloaded at: https://www.mdpi.com/article/10.3390/plants15172697/s1, Table S1: GenBank accession numbers and geographic origin of the *Apscaviroid nanocitri* (citrus dwarfing viroid, CDVd) strains and reference sequences used in the phylogenetic analysis.
