# Supplementary material for: Occurrence and Characterization of Graft-Transmissible Pathogens of Citrus in Orchards and Urban Citrus in Chile with a Focus on the Apscaviroid nanocitri
Source: Plants (Basel). 2026 Sep 2;15(17):2697. doi: 10.3390/plants15172697 (PMC13567996; doi:10.3390/plants15172697)
Supplement: Supplementary file 1 [file plants-15-02697-s001.zip › plants-4432417-supplementary.pdf]

Supplementary material

Table S1: GenBank accession numbers and geographic origin of the *Apscaviroid nanocitri* (citrus dwarfing viroid, CDVd) strains and reference sequences used in the phylogenetic analysis.

Isolate	Country	GenBank code
P1	Chile, Santiago	PZ470503.1
P21	Chile, Santiago	PZ462916.1
P28	Chile, Santiago	PZ496678.1
P37	Chile, Santiago	PZ496679.1
0001CDVd	Argentina	OK181215
0002CDVd	Argentina	OK181216
2209CDVd	Argentina	OK181217
H2-2	Italy	JF970266.1
H20-3	Italy	EU938650.1
H10-7	Italy	JF970268.1
H20-7	Italy	JF970270.1
CR-1	Italy	EU938644.1
ES-13	Italy	EU938646.1
PR-7	Italy	EU938643.1
H6-2	Italy	EU938647.1
962-3	Spain, Valencia	EU934014.1
612-2	Spain, Valencia	EU934025.1
E822	USA, California	EU934031.1
S76452 (CDVd IIIa)	USA	S76452.1
AF184147 (CDVd IIIb)	USA, California	AF184147.1
AF184149 (CDVd IIIc)	USA	AF184149.1
L85	Laos, Champasak	MT917194.1
CBLVd	Israel	NC_001651.1
